# Feasibility study on the use of elemental profiles to authenticate aromatic rice: the case of Basmati and Thai rice

**DOI:** 10.1007/s00216-021-03455-9

**Published:** 2021-06-22

**Authors:** Catalina Dumitrascu, Yiannis Fiamegos, Maria Beatriz de la Calle Guntiñas

**Affiliations:** 1 European Commission, Joint Research Centre (JRC), Retieseweg 111, 2440 Geel, Belgium; 2 Present Address: Antwerp University, Campus Drie Eiken, Universiteitsplein 1, D.S.552, 2610 Wilrijk, Belgium; 3 Present Address: Research Executive Agency, European Commission, Place Rogier 16, 1210 Brussels, Belgium

**Keywords:** Rice, Basmati, Thai, Elemental profile, Energy-dispersive X-ray fluorescence (ED-XRF) spectroscopy, Multivariate analysis

## Abstract

Among the thousands of existing rice varieties, aromatic rice has increasingly attracted consumer’s preference in recent years. Within aromatic rice, Basmati, cultivated in some regions in Pakistan and India, is highly demanded. Other aromatic rice, cultivated in specific regions, for instance in Thailand (commonly referred to as Jasmine Thai rice), are also highly appreciated by consumers. In this work, the elemental profiles of commercially available rice samples (17 Basmati, 11 Thai, and 7 Long Grain rice) were determined by energy-dispersive X-ray fluorescence (ED-XRF) spectroscopy. The mass fractions of P, Cl, S, K, Fe, Cu, and Zn were significantly different (95% confidence interval) between Basmati and Thai rice and between Thai and Long Grain rice; only Cl, S, and Zn were significantly different between Basmati and Long Grain rice. Multivariate evaluation of the results combining soft independent modelling by class analogy (SIMCA) and partial least square discriminant analysis (PLS-DA) allowed the correct classification (true positives) of 94.1, 85.6, and 100% of the Basmati, Long Grain, and Thai rice, respectively. The specificity (true negatives) of Basmati, Long Grain, and Thai was 94.4, 82.1, and 100%, respectively.

## Introduction

Rice is the most important food crop in the world [1] and staple food for more than half the world’s population [2]. Consumer’s interest in aromatic rice has increased in recent years, in particular for some types. Basmati rice is a long grain rice whose traditional varieties, grown in the Punjab provinces of Pakistan and India and the regions of Uttar Pradesh and Haryana of the same two countries, may reach prices five times higher than non-Basmati rice [3]. Basmati rice is susceptible to diseases, can only grow some periods of the year because it is photoperiod sensitive, and has tall culms [4]. To overcome these problems, many attempts were made to produce new Basmati varieties, some of them not successful in maintaining the appreciated organoleptic characteristics of the traditional Basmati [1, 5]. Recently, India has applied for the registration of Basmati rice as a protected designation of origin (PDO)/protected geographical indication (PGI) product in the quality schemes for agricultural products and foodstuffs of the European Commission [6].

Basmati is not the only rice variety that scores high in consumer appreciation; high-quality Thai jasmine rice, Khao Dawk Mali 105, grown in the Thung Kula Rong Hai area, is the first product from Southeast Asia holding a geographical indication (GI) label [2] and is also recognised as high-quality rice, widely consumed throughout the world.

The higher prices that consumers are willing to pay for some premium rice may prompt fraudulent activities, such as mislabelling lower quality grains of different botanical variety or geographical origin. To fight fraud, analytical methods that can be used in authentication studies by control laboratories to characterise the genuine products are needed.

DNA analysis is commonly used to classify rice according to its botanical variety [4] and many countries request an authentication certificate based on DNA analysis; in India, a full DNA certification process has been put in place by official bodies [5]. Other techniques such as gas chromatography (with and without mass spectrometry detection), high-performance liquid chromatography (HPLC), and fluorescence, ultra-violet (UV), and infra-red (IR) spectroscopy have also been applied to classify Basmati rice [4], with different success rates.

DNA analyses cannot differentiate rice of a certain botanical variety cultivated in different geographical regions. Multielemental and stable isotope analysis (IRMS) are probably the most widely used approaches to authenticate the geographical origin of rice, frequently in combination with multivariate analysis [2, 7–10]. IRMS is an efficient and sensitive approach, but instrumentation is expensive and requires staff with advanced analytical skills [11]. Most control laboratories have accredited methods in place for multielemental analyses, which guarantees the quality and comparability of the results. Inductively coupled plasma-mass spectrometry (ICP-MS) is the approach most widely used to classify rice based on their geographical origin [7, 8]. However, both IRMS and ICP-MS require cumbersome sample digestion procedures and are therefore not ideal for screening purposes. Recently, some works have been published on multielemental analysis of rice by neutron activation analysis (NAA) [2, 10], which eliminates the need of sample digestion, allowing also the determination of elements, such as Cl and Br, whose quantification after sample digestion is not straightforward [12]. However, NAA requires an infrastructure (nuclear reactor) which is not available in most control laboratories.

Energy-dispersive X-ray fluorescence (ED-XRF) is a type of analysis that does not require sample digestion.The only sample treatment that is required is milling and preparation of a pellet under preassure. Determination of elements such as Cl and Br is straightforward and since the amount of sample taken for analysis is high, 5 to 7 g, heterogeneity issues are of little concern in ED-XRF analyses. ED-XRF instruments are available as hand-held and portable devices, making possible in situ analysis. The main drawback of ED-XRF in comparison with ICP-based methods is the higher limits of quantification (LOQs), which are at the low mg kg^**−**1^ level for most elements. A way to decrease the LOQ in ED-XRF is to increase the irradiation time of the sample, but of course sample throughput needs to be considered, certainly in the frame of control analysis. ED-XRF has been successfully used to classify honey samples based on geographical origin and on botanical variety [13, 14]. The mass fractions of most elements are frequently lower in honey than in rice [2, 15], and therefore the ED-XRF LOQs should be low enough for the analysis of a number of elements in rice.

In this study, 35 commercially available long grain rice samples (17 Basmati, 11 Thai, and 7 Long Grain, not belonging to any of the other two types) were analysed by ED-XRF, to evaluate if it is feasible to differentiate the three mentioned rice types based on their elemental profiles, what could be of great interest for control laboratories. Multivariate analysis, principal component analysis (PCA), soft independent modelling by class analogy (SIMCA), and partial least square discriminant analysis (PLS-DA) were used to exploit the information content of the whole element profile provided by ED-XRF analysis.

## Materials and methods

### Rice samples

Thirty-five rice samples, all of them purchased at retailers in different European countries (Austria, Belgium, France, The Netherlands, and Spain), were included in the study. The set consisted of 17 Basmati, 11 Thai, and 7 Long Grain (LG) rice samples. The labels of the 7 LG samples did not contain a reference to Basmati or Thai rice. According to the information on the labels, 4 Basmati samples were a mixture of rice from India and Pakistan, 3 came from India, and other 2 from Pakistan. For 1 sample, the label indicated Himalaya as the region of origin, and no information about geographical origin was available for the remaining 6 Basmati rice samples. Nine of the Thai rice samples clearly indicated that the country of origin was Thailand, and no information on geographical origin was available for the remaining two. One LG sample came from India, 1 sample from Pakistan, 1 was a mixture of rice from India and Pakistan, and no information about geographical origin was available for the remaining 4.

While all Basmati rice samples clearly indicated the name Basmati on the label, the nomenclature for the Thai samples was not so uniform, 3 samples indicated Jasmine rice, 1 Thai Jasmine, 1 Pandan rice, and 6 either just called the rice ‘Thai’ or only indicated that the rice came from Thailand.

All rice samples included in the study were purchased in the period 2018 to 2020.

Table 1 summarises all the information included in the labels of the samples analysed. It also compiles the mass fractions obtained with the described method for the elements analysed, and information about LOQs and expanded associated uncertainties (k=2).

**Table 1:** Information on the rice samples included in the study and mass fractions of the elements analysed

Sample id	Type	Origin	Purchased in	P	Cl	S	K	Ca	Mn	Fe	Cu	Zn	Br	Rb	Ba
LOQ (mg kg^−1^)				171	78	700	566	118.4	2.55	4.6	1.2	5.8	1.7	4.2	2.4
U (k=2) (%)				6	2	10	3	3.5	11	6.5	10.5	6.5	22	5	18
RICE0001	Basmati	NA	Belgium	1162	430.4	1374	1040		8.47	7.50	1.67	16.00			
RICE0002	Basmati	India and Pakistan	Belgium	1678	216.7	1471	1164	268.7	7.49	14.02	2.01	21.20	44.12		2.46
RICE003	Basmati	NA	Belgium	1178	240.7	1612	795.4		8.75	6.63	1.83	18.39	3.14		7.66
RICE004	Basmati	India	Belgium	976.3	407.2	1412	934.7		8.91	5.62	1.58	15.15			
RICE005	Basmati	NA	Belgium	1433	455.6	1370	1129		9.94	5.81	1.24	15.77			6.95
RICE006	Basmati	NA	Belgium	1149	535.8	1509	924.8		8.48		2.06	16.79			2.49
RICE007	Basmati	Himalaya	Belgium	1277	174.0	1485	781.7		8.70		1.60	17.69		4.54	
RICE008	Basmati	India and Pakistan	Belgium	1847	507.6	1058	1580	254.2	10.61	9.37	1.44	16.29			33.27
RICE009	Basmati	NA	UK	1054	516.2	1441	989.5		7.79	7.58	1.91	16.74			
RICE0010	Basmati	India	Belgium	1452	463.6	1389	1186		9.77	6.01	1.29	16.06			
RICE0011	Basmati	India	Belgium	1277	249.8	1511	797.3		8.79	4.87	2.41	18.13			
RICE0012	Basmati	Pakistan	Austria	1017	290.5	1234	1214		5.57		1.38	11.31	15.35		
RICE0013	Basmati	NA	UK	1302	255.9	1350	958.1		7.87		1.72	15.11	41.10		2.66
RICE0014	Basmati	India and Pakistan	Belgium	1639	324.6	1358	1185		8.14	5.63	1.60	14.95	4.39		8.66
RICE0015	Basmati	Non-EU	The Netherlands	1716	491.5	1194	1416		10.12	12.28	1.46	16.71			
RICE0016	Basmati	India and Pakistan	The Netherlands	1375	331.6	1365	1027	184.1	9.88	6.53	1.30	14.81			
RICE0017	Basmati	Pakistan	Spain	1047	403.5	1405	1161		8.55		1.28	15.17			
RICE0018	Thai	Thailand	Belgium	855.9	272.0	948.1	803.6		9.39			19.72		4.83	
RICE0019	Thai	Thailand	France	846.6	239.7	943.0	669.8		9.28			18.54			63.83
RICE0020	Thai *(Jasmine)	Thailand	Belgium	1009	230.6	802.2	734.6		7.96		1.22	16.70			
RICE0021	Thai *(Jasmine)	Thailand	Belgium	906.6	261.8		739.5		9.80		1.51	17.92		5.56	41.46
RICE0022	Thai (Jasmine)	Thailand	Belgium	865.8	347.3	862.5	740.2		9.28			19.46	49.11		
RICE0023	Thai	Thailand	Belgium	1076	221.8	1073	853.9		8.88		1.26	16.22			
RICE0024	Thai	NA	UK	905.2	123.9	1209	656.1		7.47		1.56	19.19			10.62
RICE0025	Thai *(Jasmine)	NA	Spain	794.2	209.3	929.0	680.0		11.09		1.70	18.57		8.65	
RICE0026	Thai	Thailand	Belgium	847.8	422.5		838.5		8.14		1.61	19.81		7.33	
RICE0027	Thai *(Pandan)	Thailand	Belgium	852.6	321.9	860.1	749.9		8.36			17.61			
RICE0028	Thai	Thailand	Belgium	789.4	291.6	1065	754.4		7.66			16.54			
RICE0029	Long Grain	NA	UK	1678	190.0	1189	1580	672.7	5.95	7.30	2.28	7.87		9.51	
RICE0030	Long Grain	Pakistan	Belgium	906.2	403.7	1405	736.8		6.34		1.32	13.06			
RICE0031	Long Grain	NA	Belgium (UK)	1264	157.6	996.7	730.2		12.62		2.25	15.08	7.57	13.10	
RICE0032	Long Grain	India and Pakistan	Spain	1593	262.7	1401	1525		2.60	5.72	2.52	8.14	55.16		
RICE0033	Long Grain	NA	Spain	1300	212.5	1215	876.2		7.61	4.95		19.55			62.15
RICE0034	Long Grain	India	Austria	951.0	260.3	1109	829.8		10.21		1.77	14.10		8.81	
RICE0035	Long Grain	NA	Austria	1240	217.4	1017	1053		8.52		1.83	17.23			

Empty cells: mass fractions < LOQ of the method. *Only the name between brackets was provided on the label

### Reagents and standards

CEREOX® wax (Fluxana GmbH, Bedburg-Hau, Germany) was added to the rice flour obtained after milling, to avoid crumbling of the pellet during the measurements.

The reference sample FLX-S13 (Fluxana, Bedburg-Hau, Germany) was used to check the performance of the ED-XRF instrument.

Deionised water used to clean the sample processing material to avoid cross-contamination between samples was obtained with a Milli-Q Plus system (*>* 18.3 MΩ) (Millipore, Billerica, MA, USA).

The calibration curves for the determination of macro- and trace elements were constructed with 45 certified reference materials (CRMs) and reference materials (RMs); 23 of them had an organic matrix. The organic CRMs and RMs used for calibration purposes were GSV1 and GSV2 (bush leaves), GSV3 (poplar leaves), GSV4 (tea leaves), GSH1 (human hair), NBS 1571 (orchard leaves), NBS1572 (citrus leaves), OBTL-5 (tobacco), BCR-129 (hay powder), ERM-CD-281 (ray grass), ERM-CD-200, NMIJ CRM7405-a, IAEA-392, IAEA-413 (algae), NIST 1570a (spinach leaves), NIST 1575a (pine needles), NIST 1567b (wheat flour), BCR-482, IAEA-336 (lichen), BCR-679 (white cabbage), and IAEA-359 (cabbage).

To evaluate the accuracy of the method, 25 CRMs and RMs were used, 13 out of which of organic matrix. The organic CRMs and RMs used were BRAN-1 (corn bran), DUWF-1 (durum wheat flour), SOWW-1 (soft winter wheat flour), NIST 1568b (rice flour), BCR-191 (brown bread), ERM-CE 2781K (mussel tissue), ERM-BD150 (skimmed milk), PVTL-6, RT3, RT5, AJJA17 (tobacco), ERM-CE464 (tuna fish), and IMEP-119 (vegetable feed).

Table 2 summarises the results of the accuracy studies carried out with cereal CRMs.

**Table 2:** Evaluation of accuracy of the ED-XRF method on cereal CRMs. All mass fractions in mg kg^**−**1^

Element	NIST-1568b (rice)			BRAN-1 (corn bran)			DUWF-1 (durum wheat flour)			SOWW-1 (soft winter wheat flour)		
	Cert.	ED-XRF	Rec. (%)	Cert.	ED-XRF	Rec. (%)	Cert.	ED-XRF	Rec. (%)	Cert.	ED-XRF	Rec. (%)
Br	8.31 ± 0.61	8.76 ± 1.93	105.4	*2.3*	*1.93 ± 0.42*	*83.9*	*6.60*	*6.41 ± 1.41*	*97.1*			
Ba				2.4 ± 0.52	2.74 ± 0.49	114.3						
Ca	118.4 ± 3.1	139.3 ± 4.88	117.7	420 ± 38	409.4 ± 14.33	97.5	278 ± 26	274.5 ± 9.61	98.8	240 ± 23	247.7 ± 8.67	103.2
Cl	301.1 ± 3.8	266.8 ± 5.34	88.6				*680*	*601.0 ± 12.02*	*88.4*	*640*	*577.0 ± 11.54*	*90.3*
Cu	2.35 ± 0.16	2.32 ± 0.24	98.7	2.47 ± 0.4	2.82 ± 0.30	114.3	4.30 ± 0.69	3.91 ± 0.41	91.0	*1.2*	*1.40 ± 0.15*	*116.5*
Fe	7.42 ± 0.44	8.52 ± 0.55	114.9	14.8 ± 1.8	14.2 ± 0.92	95.9	41.50 ± 4	38.24 ± 2.49	92.1	*29 ±*	*16.00 ± 1.04*	***56.0***
K	1282 ± 11	1366 ± 40.98	106.6	566 ± 75	529.0 ± 15.87	93.5	3180 ± 140	3119 ± 93.57	98.1			
Mn	19.2 ± 1.8	19.07 ± 2.17	99.3	2.55 ± 0.29	2.17 ± 0.24	85.1	16.00 ± 1	14.02 ± 1.54	87.7	5.4 ± 0.6	4.85 ± 0.53	89.8
P	1530 ± 40	2128 ± 127.7	**139.1**	171 ± 11	202.8 ± 12.17	118.6	2900 ± 220	3109 ± 186.5	107.2	1080 ± 60	1119 ± 67.14	103.6
Rb	6.198 ± 0.026	7.08 ± 0.35	114.3									
Zn	19.42 ± 0.26	18.91 ± 1.23	97.4	18.6 ± 2.2	18.8 ± 1.22	101.1	22.20 ± 1.7	22.17 ± 1.44	99.8			

Cells in italic: mass fractions for which only indicative values were provided by the CRM producer. Empty cells: mass fractions not available or below the LOQ of the ED-XRF method. Cells in bold: recovery outside the range 80-100 %

### Instrumentation and sample preparation

The Br, Ba, Ca, Cl, Cu, Fe, K, Mn, Ni, P, Rb, S, and Zn mass fractions in the rice samples were measured with an Epsilon 5 ED-XRF spectrometer (PANalytical, Almelo, The Netherlands), using a previously validated method. The method is described in detail elsewhere together with the outcome of the validation study and its performance characteristics [16]. Summarising, calibration curves for quantification purposes were built up with 45 certified reference materials (CRMs), 23 of them organic matrices. The method accuracy was evaluated with 21 CRMs (11 inorganic and 10 organic matrices) and 4 organic matrix reference materials, listed above.

The standard uncertainty was evaluated analysing three pellets in triplicate in one measurement session. One pellet was analysed in three different measurement sessions, with a calibration curve newly constructed in each session. The influence of different analysts and amount of sample intake was studied. The within-pellet variation was the main contribution to the uncertainty of the results, and it was larger than the uncertainty that originated from the between-pellet and between-session variations. During the measurements, pellets spin in the Epsilon 5 spectrometer; in this way, the results obtained take into consideration the mass fractions in different points of the pellet reflecting the heterogeneity within a pellet. For this reason, only one pellet per sample was measured. The associated expanded uncertainties (k=2) (Table 1) include within- and between-pellet variation calculated by analysis of variance (ANOVA) for a confidence interval of 95%.

The LOQs were calculated in an empirical way, as the mass fractions for which certain trueness and intermediate precision were achieved.

The performance of the ED-XRF spectrometer was controlled once a week as recommended by the instrument manufacturer, measuring the reference sample FLX-S13. The results of the FLX-S13 reference sample were used to correct the normal drift of the instrument. The tobacco CRM PVTL-6, with certified values for all the elements included in the study, was used to control the accuracy of the measurements on a weekly basis. No systematic bias was observed for any of the elements.

Rice samples were dried at 70 °C for 24 h and milled for 5 min in a benchtop Planetary MonoMill-Minimill II (Fritsch-PANanalytical, Almelo, The Netherlands), in a 250-mL tungsten carbide grinding bowl, using 5 balls of 30-mm diameter of the same material. Six grammes of the dried rice powder was accurately weighed and mixed with 1 g CEREOX® wax, using a metal-free spatula. To evaluate if the CEREOX® wax contained quantifiable amounts of any of the measured elements, one pellet was made using exclusively wax. No element could be quantified, and hence blank corrections were not applied. Forty-millimetre-diameter pellets were made using aluminium cup dies, pressing the sample under 200 kN for 3.5 min with a semi-automatic press from Herzog Maschinenfabrik Gmbh (Osnabrück, Germany).

All equipment used, from grinding to preparation of the pellets, is carefully rinsed between two samples using distilled water, to avoid cross-contamination.

One pellet was made for each sample and each pellet was measured once.

### Statistical tools and multivariate analysis

The Student’s t-test (95% confidence interval), run to identify the elements (variables) whose mass fractions were significantly different in the Basmati, Thai, and Long Grain rice groups, respectively, was carried out with the software Statistica (TIBCO, version 13.5.0.17).

Multivariate analysis of the data was carried out with the software SIMCA version 15.0.2 (Sartorius Stedim Biotech AS, Malmö, Sweden) [17].

Principal component analysis (PCA), a non-supervised technique, and partial least square discriminant analysis (PLS-DA), a supervised technique that maximises the differences between two groups, were used to visually evaluate if the analysed rice form different clusters, corresponding to Basmati, Thai, and Long Grain rice, respectively. PLS-DA and soft independent modelling by class analogy (PCA-Class), a supervised technique that maximises the similarities among the observations within one group, were used for classification purposes.

In all multivariate studies, the amount of principal components was kept to three to avoid overfitting.

The presence of outliers in any of the three studied groups was evaluated with the Mahalanobis distance (DModX PS+), which is the distance between a point and the centroid of the distribution. A sample was considered an outlier when its Mahalanobis distance was larger than D_crit_ (95% confidence interval). None of the samples included in this study was flagged as outlier, and hence none was removed for further statistical analysis.

DModX PS+ was also used to study the false positive (FP) and false negative (FN) rates in each model. False positives are samples not flagged as outliers by the models although they do not belong to the targeted group, and false negatives are samples flagged as outliers although they belong to the targeted group. When the rate of false negatives is high, the sensitivity of the model is poor, while a high rate of false positives is linked to poor specificity. The proportion of correct classifications (true positives, TP, and true negatives, TN) is referred to as the accuracy of the models. The rate of TP, TN, FP, and FN of the different models was assessed by cross-validation, leaving each time one sample out and using the resulting model to classify the left out sample. The sensitivity, specificity, and accuracy of the models constructed were calculated according to Barbosa et al. [18].

Rice samples were classified following a two-step approach. First, samples were classified using PCA-Class, which is the term used by the software for SIMCA. When a sample was allocated into more than one class, it was classified as belonging to the one with the highest probability of class membership. When the highest probability of class membership for a certain sample does not correspond to the group to which the sample belongs according to the label information, the sample is considered a false negative. False negatives were classified in a second step using a PLS-DA model constructed for two groups: the group to which the sample belongs according to the information on the label and the group indicated by PCA-Class as the highest probability of class membership. This approach has been successfully applied to the classification of Spanish PDO honeys of different geographical and botanical origin [14].

## Results and discussion

### Elemental mass fractions in Basmati and Thai rice determined by ED-XRF

The mass fractions obtained by ED-XRF in Basmati and Thai rice are in good agreement with results already published in the literature, mostly obtained by ICP-MS [2, 19, 20].

More than half of the Basmati, Thai, and Long Grain rice samples had Ba, Br, Ca, Ni, and Rb mass fractions below the LOQs of the method and therefore were not included in further statistical evaluation of the data (Table 1). The Fe content was below the LOQ in more than 50% of the Thai rice samples; however, more than 50% of the Basmati and Long Grain rice had quantifiable amounts of Fe, and for that reason, Fe was included in uni- and multivariate statistical studies.

The first statistical study carried out on the results, consisted in a comparison of the mean and the median (robust location estimate not affected by extreme values) obtained for the mass fractions of the individual elements that were present in more than half the number of samples in a certain group. A good agreement between mean and median, taking into consideration their associated expanded uncertainties (95% CI), was observed for all elements with only few randomly distributed exceptions, indicating that the mass fraction results are normally distributed.

**Fig. 1:**
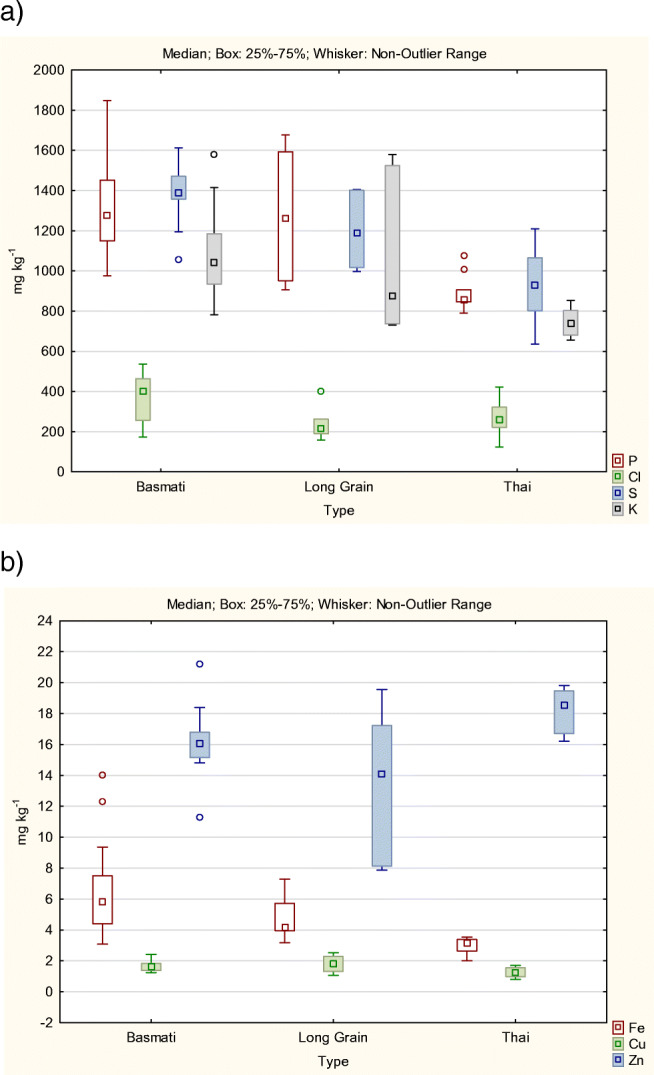
Box-whisker plot constructed with the mass fractions of (**a**) P, Cl, S, and K, and (**b**) Fe, Cu, and Zn, of the Basmati, Thai, and Long Grain rice included in the study

According to the Student’s t-test (95% confidence interval), P, Cl, S, K, Fe, Cu, and Zn were significantly different in Basmati and Thai rice and in Thai and Long Grain rice. Only Cl, S, and Zn were significantly different in Basmati and Long Grain rice. As shown in Fig. 1 a and b, Basmati and Long Grain rice are richer than Thai rice in all elements included in the study, with the exception of Zn and Cl (higher in Thai than in Long Grain). Mn could be quantified in all the rice samples analysed but the mass fractions were not significantly different in any of the three groups, and for this reason, Mn was not used for modelling purposes.

### Multivariate analysis of data

The reduced number of samples (observations) included in this study makes that its outcome can only be considered as indicative of the potential of the method. A full validation study should include 30 to 50 samples [21]. Nevertheless, as important as the amount of observation is their representativity of the wider variation that can be expected in a certain group. In this study, only commercially available samples, produced in different years and countries and commercialised by different brands and in different countries, are included. This approach warrants that the variability introduced by cultivation, production, transportation, and storage conditions is incorporated in the multivariate analysis, avoiding overfitting. Frequently, models are constructed with a high number of samples coming from one single production site, all of them produced in the same year. The traceability record is excellent but the samples are, strictly speaking, aliquots of the same cultivation batch. The standard deviation of the model would be artificially low and its performance characteristics artificially good. Any sample coming from a different region, or even production field, could become a false negative, what would lead to undue rejection of samples from the market, with the corresponding associated economic loss for the producer. With the approach used in this study, it is quite unlikely that all the commercial samples included in the study were produced in the same field and by the same producer.

**Fig. 2:**
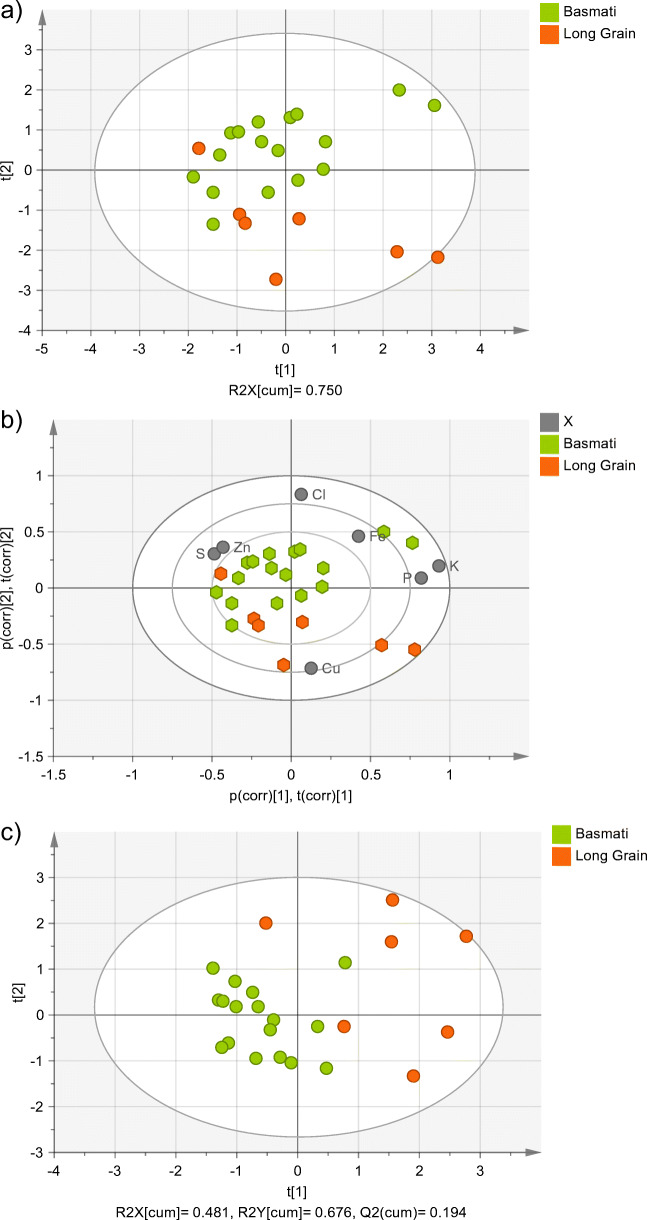
(**a**) PCA score plot (showing the two first principal components), (**b**) bi-plot of the PCA score plot, and (**c**) PLS-DA score plot (showing the two first principal components) of Basmati and Long Grain rice. Ellipse: Hotelling’s T2 (95%)

Figure 2 a shows the PCA score plot of the Basmati and Long Grain rice. The two groups are mostly separated along the second principal component. According to the PCA bi-plot (projection that overlays the information corresponding to observations and variables) (Fig. 2b), Cu and Cl are the variables mostly contributing to this separation, although the Cu mass fraction is not significantly different in the two groups according to Student’s t-test (95% confidence interval). This is the added value of multivariate analysis; PCA concentrates the information of all the used variables in some few components (three in our study), allowing discrimination that no single element mass fraction would allow. Two Basmati and two Long Grain samples are separated from the centroids of their respective groups, due to high K and P mass fractions. The PCA score plot also shows that one Long Grain sample is projected together with the Basmati rice, and one Basmati rice is projected next to the Long Grain samples. The same can be observed in the PLS-DA score plot (Fig. 2c).

**Fig. 3:**
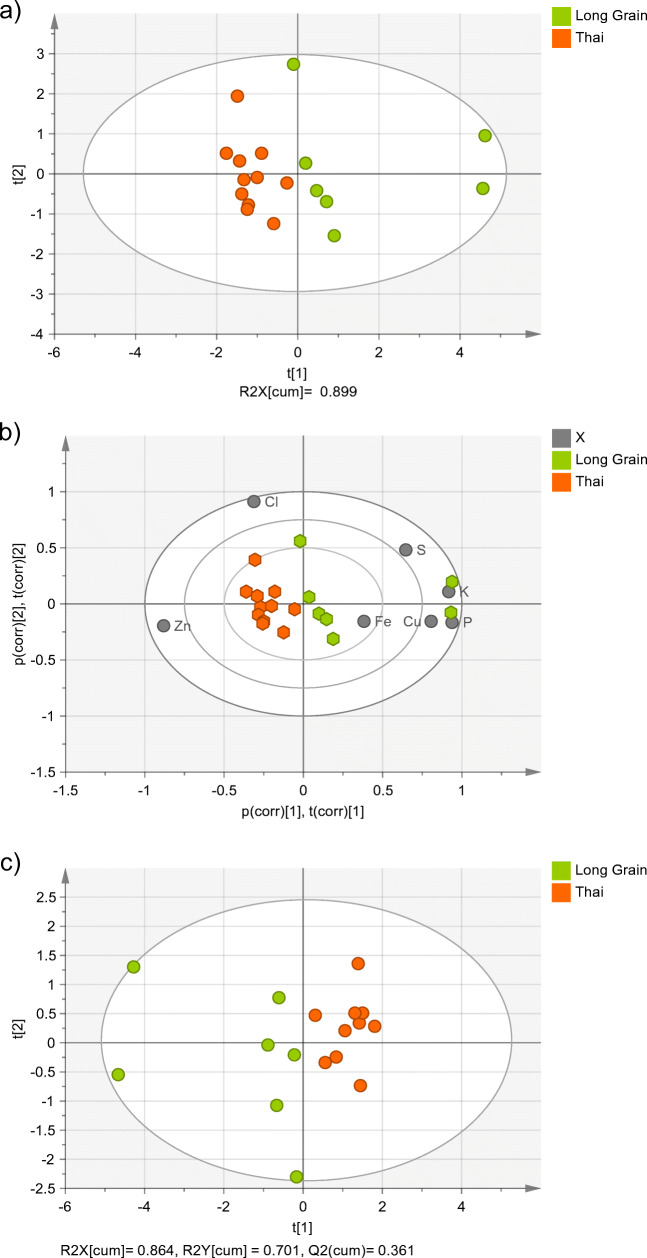
(**a**) PCA score plot (showing the two first principal components), (**b**) bi-plot of the PCA score plot, and (**c**) PLS-DA score plot (showing the two first principal components) of Thai and Long Grain rice. Ellipse: Hotelling’s T2 (95%)

Figure 3 a, the PCA score plot for the Long Grain and Thai rice, shows that the separation of these two groups takes place mostly along the first principal component axis, with two Long Grain samples clearly separated from the rest of that group. Figure 3 b shows that those two samples are particularly rich in P and K, confirming the information provided by Fig. 2 b. Thai rice is richer in Zn and Cl and both elements have a clear contribution to the separation of the Thai and Long Grain rice. The same trend was observed in the PLS-DA score plot (Fig. 3c).

**Fig. 4:**
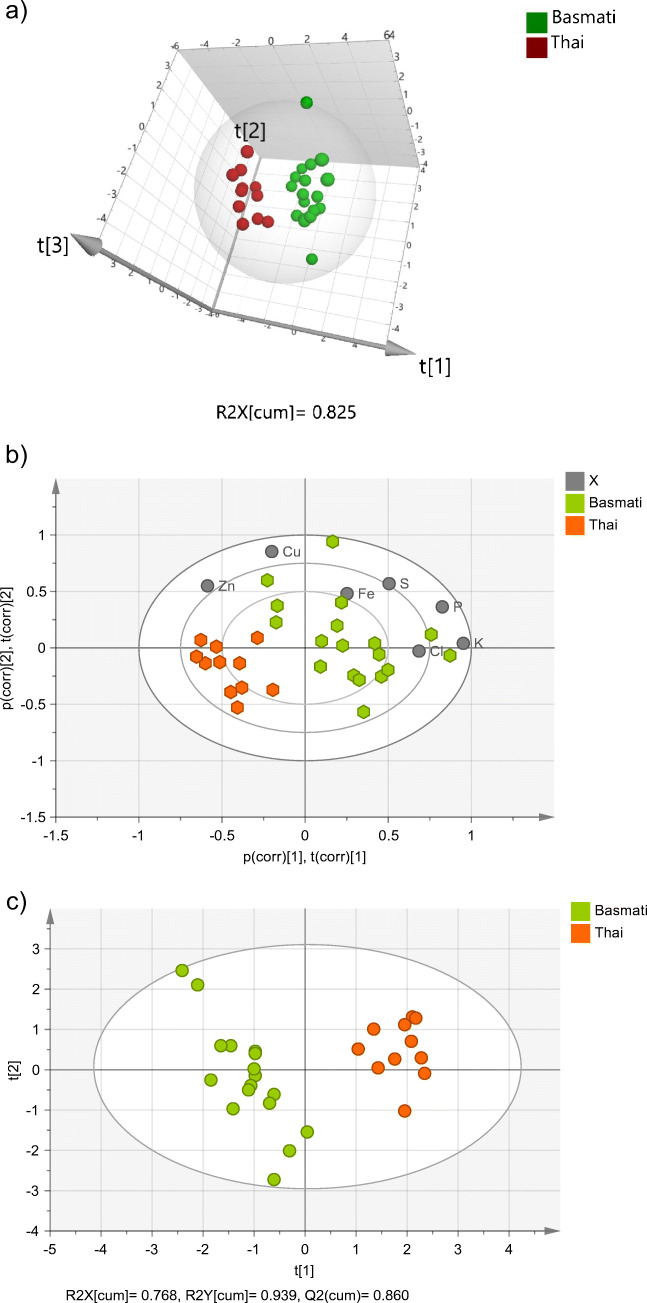
(**a**) PCA score plot (showing the three first principal components), (**b**) bi-plot of the PCA score plot, and (**c**) PLS-DA score plot (showing the two first principal components) of Basmati and Thai rice. Ellipse: Hotelling’s T2 (95%)

Basmati and Thai rice form the best separated clusters both in the PCA and PLS-DA score plots (Fig. 4a and c, respectively). This finding was expected because all Thai rice analysed were, according to the information provided on the product labels, grown in Thailand, and at least 11 of the Basmati samples were produced in India/Pakistan, two perfectly differentiated geographical regions. Three of the Long Grain rice were also grown in India and/or Pakistan, although most likely not in the same regions where Basmati rice is grown, or at least not by 100%. No information was given about the specific region of origin for the remaining Long Grain rice samples. The bi-plot of the PCA score plot confirms that Basmati rice is richer than Thai rice in all the elements used for modelling purposes, with the exception of Zn (Fig. 4b).

Table 3 summarises the performance characteristics of the two different approaches used for classification purposes, PCA-Class and PLS-DA. In a first instance, an attempt was made to classify the samples using PCA-Class, which tests if a sample belongs or not to a certain class, based on the characteristics that the observations in that class have in common. The results showed that both the Basmati and Thai rice models had a high rate of FN (samples that were either classified as Long Grain rice or could not be classified in any of the three groups). In the second approach, all samples that have been misclassified by PCA-Class, or that could not be classified at all, were classified using a PLS-DA model constructed for two groups, the one indicated on the label of the product and the one with the highest probability of class membership in PCA-Class. The rate of correct classifications increased with this approach, with only one Basmati rice classified as Long Grain rice and one Long Grain rice classified as Basmati. It is quite unlikely that a Basmati rice would be sold as Long Grain, because of the premium price paid by Basmati rice. However, with the setup used in this feasibility study (use of commercially available samples without any authenticity traceability record), it is not possible to know if the Basmati rice sample classified as Long Grain is a FN or a case of fraud. All Thai samples were correctly classified as such, and none of the Basmati or Long Grain rice samples was wrongly classified as Thai.

**Table 3:** Performance characteristics of the PCA-Class models, in terms of sensitivity, specificity, and accuracy

	Sensitivity (%) [TP/(TP + FN)]		Specificity (%) [TN/(TN+FP)]		Accuracy (%) [(TP + TN)/(TP + FN + TN + FP)]	
	PCA-Class	PCA-Class + PLS-DA	PCA-Class	PCA-Class + PLS-DA	PCA-Class	PCA-Class + PLS-DA
Basmati (n=17)	82.4 (3 FN)	94.1 (1 FN)	94.4 (1 FP)	94.4 (1 FP)	88.6	94.3
Thai (n=11)	72.7 (3 FN)	100	100	100	91.4	100
Long Grain (n=7)	85.6 (1 FN)	85.6 (1 FN)	82.1 (5 FP)	96.4 (1 FP)	82.9	94.3

## Conclusions

This feasibility study has demonstrated that elemental profiles can be used to verify the correct labelling of Asian aromatic long rice with a premium price such as Basmati and Thai rice. The mass fractions of seven elements, P, K, Cl, S, Fe, Cu, and Zn, are enough to achieve an accuracy in the classification in the range 94 to 100%. Analysis by ED-XRF does not imply tedious sample treatments and since hand-held devices are commercially available, the technique is suitable for on-site control analysis.

So far, only the potential of the method has been shown. To carry out a full validation, more samples than the ones used in this study would be needed; ideally 30 to 50 samples per class with well-documented traceability should be included. Most likely, control laboratories in the geographical regions where Basmati and Thai rice are produced would have easy access to a representative number of reference samples obtained in all the regions in which the mentioned types of rice are produced. The inclusion of commercially available samples is also recommended to cover all possible sources of variability, also different transport and storage conditions, avoiding in this way overfitting of the models used for classification purposes.

The approach could also be applied to discriminate the geographical origin of the studied rice varieties. Geographical origin and botanical varieties are the two key factors determining the elemental composition of vegetables. It was not possible to carry out such a study in this work due to the limited amount of samples with a known geographical origin.

ED-XRF can be used in studies on nutritional aspects related to the consumption of the different rice varieties; the LOQs achieved are low enough to quantify major and most trace essential elements.
